# New species of Southeast Asian Dwarf Tarantula from Thailand: *Phlogiellus* Pocock, 1897 (Theraphosidae, Selenocosmiinae)

**DOI:** 10.3897/zookeys.684.12558

**Published:** 2017-07-11

**Authors:** Narin Chomphuphuang, Deborah Smith, Sitthipong Wongvilas, Varat Sivayyapram, Chaowalit Songsangchote, Natapot Warrit

**Affiliations:** 1 Zoology Program, Faculty of Science, Chulalongkorn University, Bangkok 10330, Thailand; 2 Department of Ecology & Evolutionary Biology, University of Kansas. Lawrence, USA; 3 Spider Planet Research Center 49/201 Sukhapiban 5 Soi 45 Rd. Orngean Saimai, Bangkok 10220, Thailand; 4 Department of Biology, Faculty of Science, Chulalongkorn University, Bangkok 10330, Thailand

**Keywords:** Distribution, Mygalomorph, natural history, taxonomy

## Abstract

A new record of the tarantula genus *Phlogiellus* Pocock, 1897 from Thailand is described. Distributional data, natural history, morphological characters, and illustrations of male and female are provided. The Thai specimens belong to a new species, *Phlogiellus longipalpus* **sp. n.** The diagnosis of the new species and related species are discussed.

## Introduction

**Figure 1.**
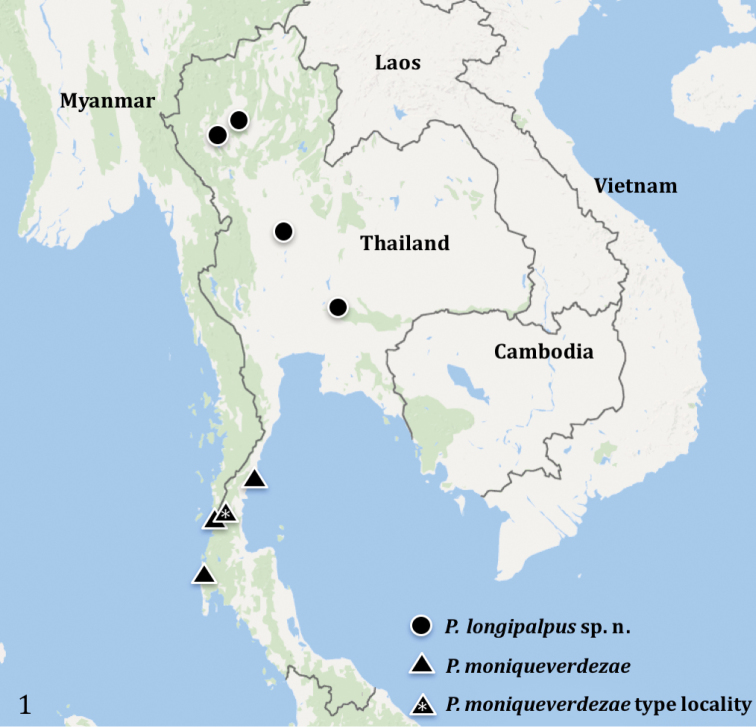
Distribution records of *Phlogiellus* in Thailand: *P. moniqueverdezae* Nunn et al., 2016 from Ranong province, and *P. longipalpus* sp. n. (Kamphaengphet, Lamphun, Lampang and Saraburi provinces).

Four genera of Theraphosidae are currently known from Thailand: *Cyriopagopus* Simon, 1887, *Ornithoctonus* Pocock, 1892, *Chilobrachys* Karsch, 1892 and *Phlogiellus* Pocock, 1897 (World Spider Catalog, 2017). *Phlogiellus*, the Asian dwarf tarantulas, was erected for *Phlogiellus atriceps* Pocock, 1897. It is mainly distributed in Southeast Asia, peninsular Malaysia, Indonesia, the Philippines, Taiwan (Orchid Island), and some islands west of Wallace’s Line (West et al. 2012, Nunn et al. 2016, World Spider Catalog 2017). *Phlogiellus moniqueverdezae* Nunn et al., 2016 is the only *Phlogiellus* heretofore reported from Thailand (Nunn et al. 2016); here we report a second Thai species, *Phlogiellus longipalpus* sp. n. Collection sites for *P. moniqueverdezae* and *P. longipalpus* sp. n. are shown in Figure 1. According to West et al. (2012) *Phlogiellus* shares the following combination of characters: number of labial cuspules between 200–350, length of posterior lateral spinnerets nearly or equal to length of metatarsus IV, and deep fovea. However, Nunn et al. (2016) recanted the use of posterior lateral spinnerets length to length of metatarsus IV as the group synapomorphic character. Kishida (1920) proposed the genus *Yamia* for some species now placed in *Phlogiellus*, citing complete lack of a lyra on the prolateral face of the maxilla as a diagnostic character. By this criterion seven species of *Phlogiellus* would be included in *Yamia*: *P. aper* (Simon, 1891), *P. brevipes* (Thorell, 1897), *P. watasei* (Kishida, 1920), *P. mutus* (Giltay, 1935), *P. bundokalbo* (Barrion & Litsinger, 1995), *P. moniqueverdezae* Nunn et al., 2016 and *P. longipalpus* sp. n. Haupt and Schmidt (2004) and Zhu and Zhang (2008) also proposed the generic status of *Yamia* but without supporting phylogenetic analyses. A cladistic analysis of the subfamily Selenocosmiinae Simon, 1889 by West et al. (2012) using a morphological data set showed monophyly of [*Phlogiellus* + *Yamia*] and did not resolve relationships among *Phlogiellus* and the putative *Yamia* species. Raven (2005) considers *Yamia* a junior synonym of *Phlogiellus*, and suggests that the maxillary lyra may be lost secondarily in *Phlogiellus* as well as other selenocosmiine genera. Here, we document a second *Phlogiellus* from central and northern Thailand and describe it as a new species, *P. longipalpus* sp. n. Illustrations of the body and copulatory organs are provided, as well as information on natural history and remarks on morphological characters distinguishing this species from previously known species.

## Materials and methods

Collections were carried out in Kamphaengphet, Lamphun, Lampang and Saraburi provinces, Thailand on 12 May 2014, 27 May 2014, 16 July 2015, and 8 Aug 2015, respectively. All tarantulas were collected and preserved in 95% ethanol. Specimens were transferred to the Center of Excellence in Entomology, Chulalongkorn University, Bangkok, for dissection and identification. All measurements were carried out using a Zeiss Stemi DV4 stereomicroscope equipped with an eyepiece micrometer. Diagnostic features were photographed using an Olympus Camedia c-4040zoom digital camera mounted to the phototube of an Olympus SZ60 stereoscope. Leg length and width measures were made on the left side of all specimens. Length of each leg segment was measured from the dorsal aspect, and leg width was measured at the basal end of the leg segment viewed from dorsal aspect. Tarsal measurements did not include claws. The relation factor (RF) was calculated as the ratio of the length of leg I to leg IV multiplied by 100 (von Wirth and Striffler 2005). Leg formula, the leg lengths in decreasing order, is also presented. Legs, pedipalps, stridulatory organs (cheliceral strikers and maxillae) were measured from the left side of all specimens. Color of morphological parts are as seen in alcohol-preserved specimens unless otherwise noted. The copulatory organs of females were dissected and cleared in 3M aqueous KOH solution. Specimens were identified by comparison of our measurements and images to those in Haupt & Schmidt (2004), Zhu and Zhang (2008), Schmidt (2010), West et al. (2012) and Nunn et al. (2016). All type and voucher specimens are deposited at the Chulalongkorn University Museum of Zoology (**CUMZ**), Bangkok, Thailand. The following abbreviations are used in the text:

**AER** anterior eye row; **AME** anterior median eyes; **ALE** anterior lateral eyes; **MOA** median ocular area; **PER** = posterior eye row; **PLE** = posterior lateral eyes; **PME** = posterior median eyes; **PLE** = posterior lateral eyes, **PLS** = posterior lateral spinnerets, **PME** = posterior median eyes, **PMS** = posterior median spinnerets, **Fem** = femur, **Pat** = patella, **Tib** = tibia, **Met** = metatarsus, **Tar** = tarsus.

All measures are given in millimeters (mm).

### Other materials

*P. moniqueverdezae* Nunn et al., 2016: 1♂ (CUMZ-T3-NA2M) and 2♀ (CUMZ-T3-NA5FM, CUMZ-T3-NA3FM), (9°46’14.2”N 98°24’44.5”E) Koh Phayam, Ranong province, Thailand; 1♂ (CUMZ-T9-COM), (8°46’17.8”N 98°16’36.0”E), Takua Pa District, Phang-nga province, Thailand; 1♀ (CUMZ-T10-COFM), (8°46’17.8”N 98°16’36.0”E), Tha Sae District, Chumphon province, Thailand (Fig. 1).

## Taxonomy

### Phlogiellus longipalpus sp. n.

Taxon classificationAnimaliaAraneaeTheraphosidae

http://zoobank.org/CF6F2F8D-15EB-48E1-BC32-7DAEA60C594D

#### Type material.

—**Thailand**: Holotype ♂, paratype 1♂, and paratype 3♀, Sai Thong Watthana district, Kamphaeng Phet province (16°17’45.6”N 99°52’49.8”E), 12^th^ May 2014. Paratype: 1♂ and 2♀, Pa Sang district, Lamphun province (18°23’46.8”N 98°51’22.2”E), 27^th^ May 2014; 1♀, Wiang Nuea district, Lampang province (18°18’09.6”N 98°30’36.6”E) 16^th^ Jul 2015, and 1 ♀, Saraburi province, Muak Lek, (14°27’27.0”N 101°11’27.0”E), 8^th^ Aug 2015. (CUMZ-(C1-NA1, C2-NA1, C4-NA2, C4-NA3, C4-NA4, C7-NA1, C8-CH2, B1-NA3, B1-NA1, B1-NA2): 3 ♂, 7♀).

#### Etymology.

The specific name refers to the Latin *longus* (“long”), which describes both male pedipalp and female spermatheca, and *palpus* (“palm of the hand” or “feeler”).

#### Diagnosis.

*Phlogiellus longipalpus* sp. n. was included in the *Yamia* group of *Phlogiellus* based on the following morphological characters: male embolus with single retrolateral keel (Fig. 19), anterior eye row slightly procurved, ALE larger than PLE (Fig. 6), clypeus narrow or absent, third claw present on tarsus IV (Haupt & Schmidt 2004; Zhu & Tso 2005). *P. longipalpus* sp. n. differs from all other *Phlogiellus* species except *P. aper*, *P. brevipes*, *P. mutus*, *P. bundokalbo*, *P. watasei*, and *P. moniqueverdezae* in lacking a maxillary lyra (Fig. 7). *P. longipalpus* differs from the latter six species in possessing a long embolus that is more or less 3 times longer than palpal bulb length (Figs 19, 21–23; Suppl. material 1, Figs A1–A8) and in the shape of the female spermatheca, which is long with an apical bend (Figs 20, 24; Suppl. material 1, Figs B1–B8). It differs from *P. brevipes* in possessing 5 spines on the posterior metatarsi (only 2 known in *P. brevipes*). It differs from *P. aper* in possessing divided scopulae on tarsus IV (Fig. 18, 28) (Nunn et al., 2016).

#### Description – Male.

**Figures 2–3.**
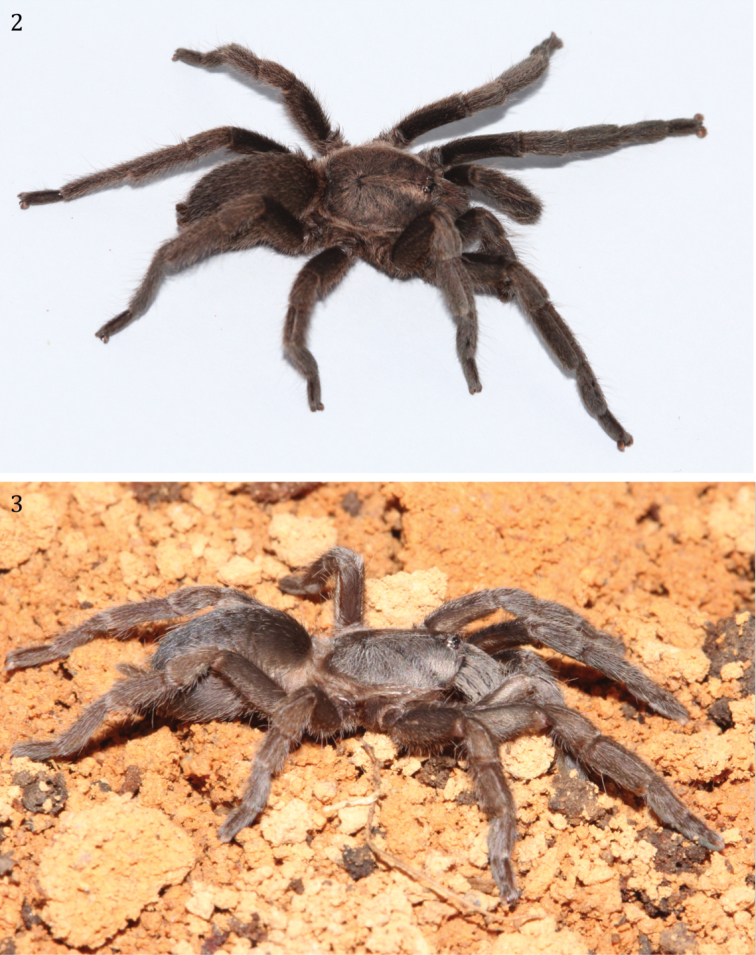
*Phlogiellus longipalpus* sp. n. **2** paratype ♂, CUMZ-C3-NA2 **3** paratype ♀, CUMZ-C4-NA4.

**Figures 4–9.**
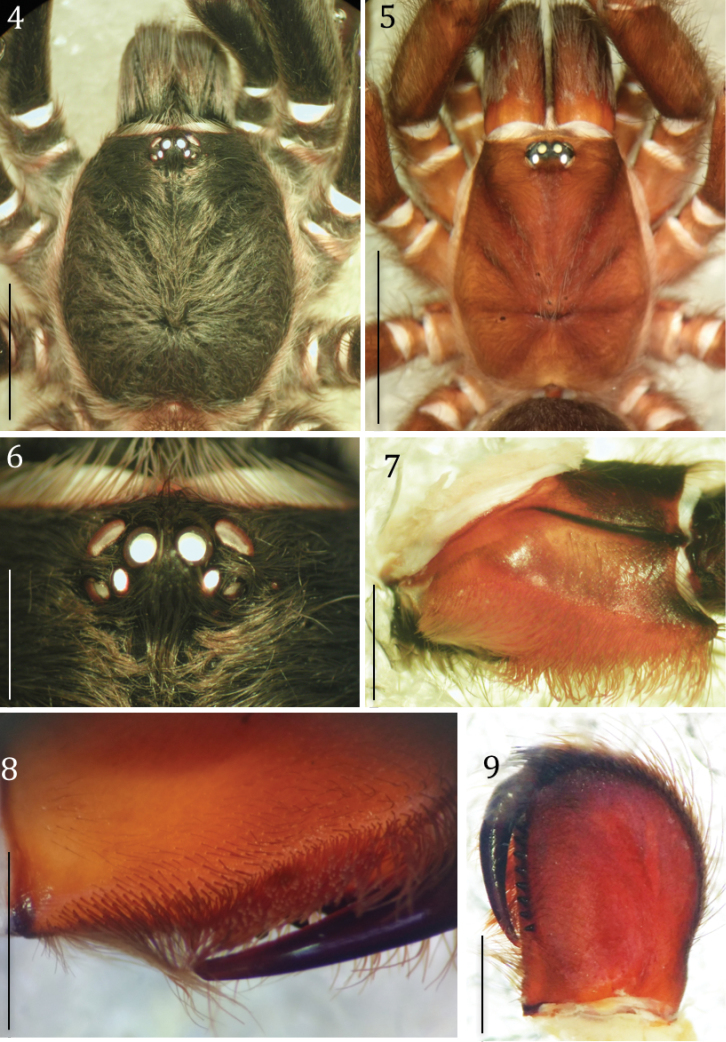
*Phlogiellus longipalpus* sp. n. **4, 6, 7** holotype ♂, CUMZ-C2-NA1: **4** carapace, dorsal view **5** carapace, dorsal view, paratype ♀, CUMZ-C4-NA4 **6** eyes, dorsal view **7** left maxilla, prolateral view. **8, 9** paratype ♂, CUMZ-C4-NA4: **8** chelicerae striker, retrolateral view **9** right chelicerae prolateral view. Scale bars: 4 mm (**4–5**); 1 mm (**6–9**).

**Figures 10–18.**
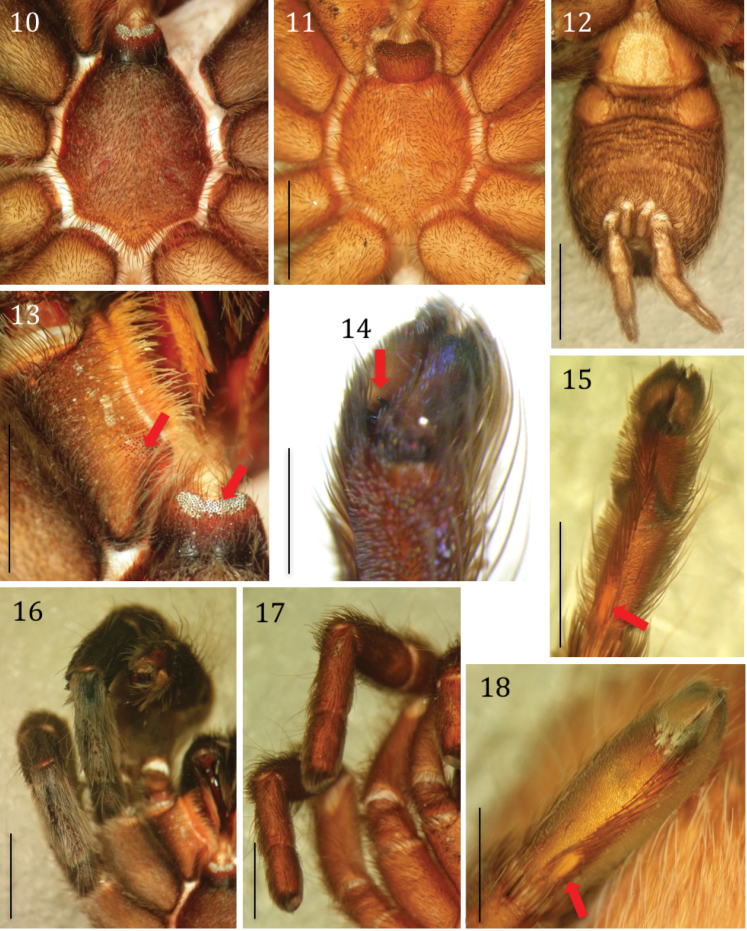
*Phlogiellus longipalpus* sp. n. **10, 12, 13, 15–16** holotype ♂, CUMZ-C2-NA1: **11–17** paratype ♀, CUMZ-C4-NA4: **14–15** paratype ♂, CUMZ-C4-NA3 **10** sternum, labium and coxae, ventral view **11** sternum, labium, and coxae, ventral view **12** abdomen and spinneret, ventral view **13** labium and maxilla, arrows indicate cuspules **14** left tarsus IV, ventral view, arrow indicates third claw **15** right tarsus IV, ventral view, arrow indicates “bald spot” **16** right legs I and II, dorsal view **17** right legs I and II, dorsal view **18** paratype ♀, CUMZ-C1-NA1, left tarsus IV, ventral view, arrow indicates “bald spot”. Scale bars: 2 mm (**10–11**); 3 mm (**12**); 2 mm (**13, 16–18**); 0.5 mm (**14**); 1 mm (**15**).

Holotype ♂ CUMZ-C2-NA1: Color (in life, Fig. 2): dark brown, carapace black. Total length (including chelicerae) 20.88; cephalothorax 8.38 long, 6.63 wide, 2.0 high (caput); fovea 1.52 wide, procurved, deep; cephalothorax black, with cover of short, whitish brown hairs dorsally, golden yellow to yellowish brown hairs on lateral margins (Fig. 4). Clypeus 0.24; ocular tubercle 0.96 long, 1.47 wide. Anterior eyes with long hairs in front of AME and mid-posterior PME area. Anterior eye row slightly procurved and posterior row slightly recurved; eyes whitish, ALE oval in shape and larger than the round AME (Fig. 6); eye lengths/widths: AME 0.30/0.28; ALE 0.39/0.27; PLE 0.24/0.18; PME 0.21/0.12; eye interdistances: AME–AME 0.21; AME–ALE 0.12; AME–PME 0.11; ALE–ALE 0.77; ALE–PME 0.21; PME–PME 0.69; PME–PLE 0.06; PLE–PLE 0.96; and ALE–PLE 0.20. Chelicerae dark, with row of 9 promarginal teeth with rows of orange-red setae (Fig. 9), a series of strikers (>60), in > 4 horizontal rows (unordered). Strongest/longest strikers on lowest rows. Each striker is needle-form (Fig. 8), lacking filiform ends. Maxillae reddish brown, 2.95 long, 1.55 wide with 115 cuspules, covered with orange-red setae on prolateral surface; maxillary lyra absent (Fig. 7). Labium blackish-brown on the basal half, reddish brown elsewhere; 0.93 long, 1.50 wide, with 202 cuspules (Fig. 13). Sternum dark-brown, covered with 2 types of hairs: strong dark and soft white (Fig. 10); 4.45 long, 3.65 wide with 3 pairs of ovoid sigillae present near lateral margins opposite coxa I, II and III. Sigilla: anterior pair obscured close to sternal margin; median pair 0.27 long, 0.15 wide 0.33 from sternal margin; posterior pair 0.42 long, 0.18 wide 0.60 from sternal margin. Abdomen 9.88 long, 6.13 wide, brownish yellow and hirsute dorsally, dark gray and thickly hirsute laterally and ventrally (Fig. 12). Legs: Pat, Tib, Met and Tar dark brown, prolateral and retrolateral surface of femora dark, thickly covered with long and short grayish white hairs (Fig. 16), coxae and trochanter dark brown dorsally, lighter brown ventrally. Met IV with 5 distal spines. Length of legs, palpal segments and RF shown in Table 1.

**Table 1.** Legs and palp measurements (in mm) of holotype CUMZ-C2-NA1 ♂ *Phlogiellus longipalpus* sp. n. Relation Factor (RF) = 99.27.

	**I**	**II**	**III**	**IV**	**Palp**
Fem	6.60	5.76	4.92	5.94	4.08
Pat	3.48	2.55	2.94	3.20	2.88
Tib	5.12	4.15	3.18	4.14	4.24
Met	3.48	3.75	3.36	4.98	-
Tar	1.83	1.83	1.83	2.40	1.51
Total	20.51	18.04	16.23	20.66	12.71

**Figures 19, 20.**
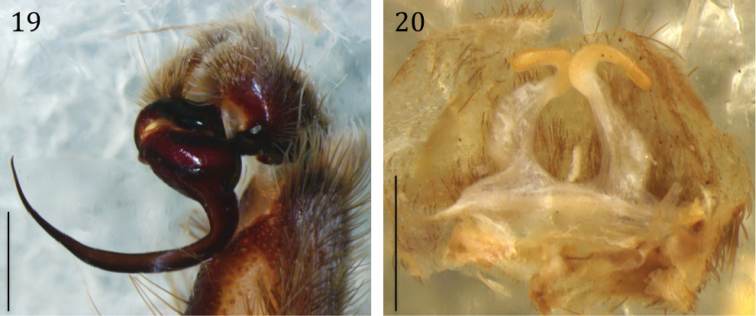
*Phlogiellus longipalpus* sp. n. **19** holotype ♂, CUMZ-C2-NA1, left pedipalps, retrolateral view **20** paratype ♀, CUMZ-C4-NA4, spermathecae, dorsal view. Scale bars: 1 mm.

**Figures 21–24.**
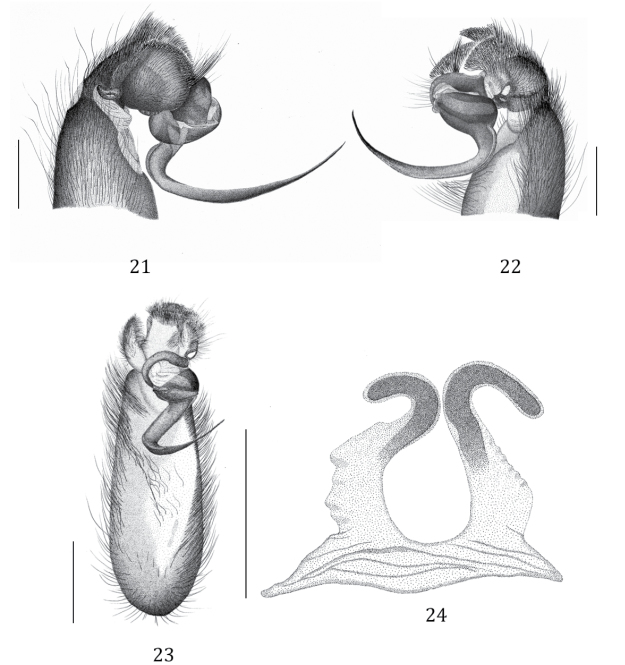
*Phlogiellus longipalpus* sp. n. **21** holotype ♂, CUMZ-C2-NA1, left pedipalp, prolateral view **22** same, retrolateral view **23** same, ventral view **24** CUMZ-C4-NA4, spermathecae, dorsal view. Scale bars 1 mm (**21–24**).

Scopulae on metatarsi and tarsi I through IV may be undivided, divided longitudinally by several rows of long, straight spiniform setae or absent. Fig. 28 illustrates diagrammatically the state of the scopulae on metatarsi and tarsi of legs I-IV for *P. longipalpus* and other *Phlogiellus* species. In addition, we noted whether the extension of the scopulae was complete (running nearly the full length of the tarsus or metatarsus) or reduced in length (e.g., extension ¾ the length of the leg segment). Scopula extension on Met I, complete; Met II, complete; Met III, complete; Met IV, ¾ and denser at distal end than proximal end. Scopula extension on Tar I, complete; Tar II, complete; Tar III, complete; Tar IV, complete but denser at the distal end, and with a small, nearly hairless oval (“bald spot”) at the proximal end. Tar II, III and IV with dense tufts of scopular hair at distal end (Fig. 15). Male tibia I spur absent. Spines: Met I and II: absent; Met III: 8 spines, Met IV: 6. Tar I–III with 2 claws, Tar IV with third claw (Fig. 14); claws covered by dense hair, dorsally with 2 rows of club-shaped setae. Spinnerets white-yellow, covered with dark longer and thinner hairs; PMS 1.16 long, 0.36 wide; PLS 4.92 long basal to apical (2.02, + 1.34, + 1.56), wide (0.64 + 0.72 + 0.41) (Fig. 12). Pedipalps dark gray reddish brown, covered with longer and thinner hairs on tibia; tibia swollen, cymbium with two lobes of light brown shaggy scopulae, bulb and embolus 3.09 long dark reddish brown (Fig. 19), palpal bulb ellipsoid and partly concave, 0.91 long, 0.51 wide; embolus extremely long, thin, curved like a partly twisted horn with sharp tip, with single retrolateral keel (Figs 21–23).

#### Variation – Male

(range (mean ± standard deviation)): Total length (including chelicerae) 13.7–21.00 (18.52±4.18); cephalothorax 6.60–8.33 (7.19±0.98) long, 2.88–6.63 (4.74±1.88) wide, 2.00–2.88 (2.51±0.46) high (caput); fovea 1.00–1.52 (1.28±0.26) wide; clypeus 0.18–0.24 (0.21±0.04) narrow or absent; ocular tubercle 0.90–1.02 (0.96±0.06) long, 1.10–1.47 (1.34±0.21) wide. Eye length/width: AME, 0.20–0.30 (0.26±0.05)/0.20–0.28 (0.25±0.04); ALE, 0.30–0.39 (0.35±0.05)/0.21–0.30 (0.26±0.05); PLE, 0.24–0.30 (0.26±0.03)/0.15–0.20 (0.18±0.03); PME, 0.15–0.21 (0.19±0.03)/0.10–0.12 (0.11±0.01). Inter-eye distances: AME–AME, 0.10–0.21 (0.16±0.06); AME–ALE, 0.10–0.12 (0.11±0.01); AME–PME, 0.10–0.12 (0.11±0.01); ALE–ALE, 0.77–0.84 (0.80±0.04); ALE–PME, 0.20–0.24 (0.22±0.02); PME–PME, 0.50–0.72 (0.64±0.12); PME–PLE, 0.06–0.10 (0.07±0.02); PLE–PLE, 0.80–0.99 (0.92±0.10); and ALE–PLE, 0.10–0.20 (0.14±0.05). Maxillae 1.70–2.95 (2.46±0.67) long, 1.2–1.56 (1.44±0.21) wide. Labium 0.90–1.14 (0.99±0.13) long, 1.20–1.74 (1.48±0.27) wide. Sternum 3.20–4.45 (3.93±0.65) long, 2.40–3.78 (3.28±0.77) wide. Abdomen 6.32–9.88 (8.32±1.82) long, 3.52–6.35 (5.33±1.57) wide. Length of legs and palpal segments shown in Table 2. Spinnerets: PMS 0.80–1.23 (1.06±0.23) long, 0.30–0.39 (0.35±0.05) wide; PLS 3.40–4.92 (4.15±0.76) long basal to apical (1.30–2.02 (1.65±0.36), + 0.90–1.34 (1.08±0.23), + 1.20–1.56 (1.43±0.20)), wide (0.39–0.64 (0.55±0.14) + 0.51–0.72 (0.59±0.11) + 0.39–0.42 (0.40±0.02)).

**Table 2.** Legs and palp measurements of ♂ *Phlogiellus longipalpus* sp. n. (n= 3) (range, mean ± standard deviation).

	**I**	**II**	**III**	**IV**	**Palp**
Fem	4.60–7.36 (6.19±1.43)	4.00–6.40 (5.39±1.24)	3.30–4.92 (4.34±0.90)	4.20–6.88 (5.67±1.36)	2.50–4.47 (3.68±1.04)
Pat	3.20–4.32 (3.67±0.58)	2.55–3.76 (2.97±0.68)	2.3–3.06 (2.77±0.41)	2.60–3.60 (3.13±0.50)	2.10–2.88 (2.58±0.42)
Tib	4.00–5.58 (4.90±0.81)	3.30–4.56 (4.00±0.64)	2.30–3.18 (2.87±0.49)	2.80–5.52 (4.15±1.36)	3.10–4.24 (3.81±0.62)
Met	3.00–4.38 (3.62±0.70)	2.80–4.14 (3.56±0.69)	2.50–3.84 (3.23±0.68)	3.90–5.76 (4.88±0.93)	–
Tar	1.70–2.58 (2.04±0.48)	1.80–2.34 (1.99±0.30)	1.83–2.40 (2.08±0.29)	2.30–2.68 (2.46±0.20)	1.30–1.51 (1.42±0.11)
Total	16.50–24.22 (20.41±3.86)	14.50–21.20 (17.91±3.35)	12.40–17.22 (15.28±2.55)	15.80–24.44 (20.30±4.33)	9.00–12.75 (11.49±2.15)

#### Description – Female.

Paratype ♀ CUMZ-C4-NA4: Color (in life, Fig. 3): dark brown, carapace brown. Total length (including chelicerae) 17.51; cephalothorax 6.56 long, 4.25 wide 2.12 high (caput); fovea 0.87 wide, procurved, deep; cephalothorax brown, covered with short whitish brown hairs dorsally, golden yellow to yellowish brown hairs on lateral margins (Fig. 5); clypeus 0.15 high; ocular tubercle 0.72 long, 1.14 wide. Anterior eyes with long hairs in front of AME and mid-posterior PME area; anterior eye row slightly procurved and posterior row slightly recurved. Eyes whitish, ALEs oval in shape, larger than the round AMEs. Eye length/width: AME, 0.30/0.15; ALE, 0.21/0.22; PLE, 0.27/0.15; PME, 0.20/0.13; Inter-eye distances: AME–AME, 0.14; AME–ALE, 0.11; AME–PME, 0.09; ALE–ALE, 0.57; ALE–PME, 0.16; PME–PME, 0.48; PME–PLE, 0.05; PLE–PLE, 0.66; and ALE–PLE, 0.11. Chelicerae dark orange with row of 10 promarginal teeth, cheliceral face with stridulatory ridges and rows of orange-red setae, a series of strikers (>60), in > 4 horizontal rows (unordered). Strongest/longest strikers on lowest rows. Each striker needleform, lacking filiform ends (Fig. 8). Maxillae reddish brown, 2.34 long, 1.24 wide with 152 cuspules, prolateral surface of maxilla covered with orange-red setae, and maxillary, and maxillae lyra absent. Labium reddish brown, 0.88 long, 1.28 wide with 271 cuspules. Sternum brownish, covered with 2 types of hair: strong dark and soft white (Fig. 11); sternum 2.9 long, 2.64 wide, with 3 pairs ovoid sigillae present near lateral margins opposite coxa I, II and III. Sigillae: anterior pair obscured close to sternal margin; median pair 0.24 long, 0.15 wide, 0.12 from sternal margin; posterior pair 0.30 long, 0.17 wide, 0.39 from sternal margin. Abdomen 9.50 long, 6.20 wide, gray-yellow and hirsute dorsally, brownish gray and thickly hirsute laterally and ventrally. Legs brownish, thickly covered with grayish white short and long hairs (Fig. 17), coxae and trochantera brown. Met IV with 5 distal spines. Length of legs, palpal segments shown in Table 3. Leg formula (length) IV, I, II, III.

**Table 3.** Legs and palp measurements (in millimeters) of paratype CUMZ-C4-NA4 ♀ *Phlogiellus longipalpus* sp. n. from Thailand. RF = 97.54.

	**I**	**II**	**III**	**IV**	**Palp**
Fem	4.48	3.35	2.95	4.15	2.65
Pat	2.40	2.10	2.15	2.20	2.10
Tib	2.75	2.10	1.75	2.80	1.90
Met	1.90	1.45	1.50	2.82	-
Tar	1.55	1.20	1.15	1.44	1.65
Total	13.08	10.20	9.50	13.41	8.30

Scopulae: Met I, II, III, undivided; Met IV, divided. Tar I, II, undivided; Tar III, IV, divided by several rows of long spines (Fig. 18). Met I, II, III, complete; Met IV, extension ¾, denser at distal end than at proximal end. Scopula extension on Tar I, II, III, IV complete; Tar IV scopula denser at the distal end and with a small, nearly hairless oval (“bald spot”) at the proximal end (see Fig. 18). Hairs of distal scopula on Tar II, III and IV more evenly distributed than in the male, not forming tufts of hair (compare Figs 15, 18). Spines: Met I and II, absent; Met III, 7 spines; Met IV, 6 spines. Tar I–III with 2 claws, Tar IV with third claw, 2 dorsal rows of club-shaped setae. Spinnerets white-yellow, covered with long, thin dark hairs; PMS 0.87 long, 0.48 wide; PLS 3.48 length of segments (from basal to apical) (1.38 + 0.87 + 1.23), width of segments (basal to apical) 0.63 + 0.54 + 0.45. Genitalia: epigastric fold 1.86. Spermathecae (Figs 20, 24): paired, each 0.3 mm wide at base and the pair fused at the base, 1.05 (left) and 1.11 (right) mm long, 0.42 (left) – 0.54 (right) wide, and apically bent; sclerotization heaviest apically, gradually decreasing basally.

**Variation**

#### – Female

(N = 7; range (mean ± standard deviation)): Total length (including chelicerae) 14.30–26.75 (20.31±4.72); cephalothorax 6.56–10.70 (8.80±1.54) long, 4.25–8.20 (6.27±1.41) wide, 2.12–4.16 (3.05±0.69) high (caput); fovea 0.87–1.68 (1.29±0.28) wide; clypeus 0.15–0.30 (0.21±0.05) or absent; ocular tubercle 0.70–1.14 (0.91±0.20) long, 1.00–1.80 (1.44±0.30) wide. Eye length/width: AME 0.20–0.40 (0.28±0.07)/0.15–0.40 (0.26±0.08); ALE 0.21–0.50 (0.36±0.10)/0.20–0.45 (0.28±0.08); PLE 0.27–0.45 (0.33±0.05)/0.15–0.20 (0.19±0.02); PME 0.20–0.40 (0.28±0.07)/0.13–0.20 (0.18±0.03). Inter-eye distances: AME–AME 0.14–0.50 (0.33±0.14); AME–ALE 0.10–0.50 (0.22±0.15); AME–PME 0.09–0.50 (0.23±0.16); ALE–ALE 0.57–1.70 (0.98±0.38); ALE–PME 0.16–0.40 (0.28±0.11); PME–PME 0.48–1.80 (0.88±0.44); PME–PLE 0.05–0.30 (0.14±0.09); PLE–PLE 0.66–1.90 (1.09±0.39); and ALE–PLE 0.11–0.50 (0.28±0.16). Maxillae 2.30–3.35 (2.73±0.41) long, 1.20–2.20 (1.66±0.39) wide. Labium 0.88–1.40 (1.06±0.20) long, 1.28–1.96 (1.50±0.27) wide. Sternum 2.90–4.82 (3.93±0.85) long, 2.64–4.32 (3.50±0.73) wide. Abdomen 7.38–15.62 (10.96±2.99) long, 4.80–8.80 (6.74±1.67) wide. Length of legs and palpal segments shown in Table 4. Spinnerets: PMS 0.80–1.40 (1.04±0.26) long, 0.40–0.60 (0.53±0.07) wide; PLS 3.48–5.13 (4.09±0.55) long from base to apex, basal segment 1.20–2.10 (1.53±0.29), median segment 0.87–1.59 (1.17±0.24), apical segment 0.90–1.89 (1.40±0.35) long; width of basal segment 0.63–1.05 (0.82±0.17), median segment 0.54–0.99 (0.75±0.16) and apical segment 0.45–0.75 (0.58±0.12).

**Table 4.** Legs and palp measurements [range (mean ± standard deviation)] of ♀ *Phlogiellus longipalpus* sp. n. (n=7).

	**I**	**II**	**III**	**IV**	**Palp**
Fem	3.20–6.90 (3.63±1.34)	3.20–5.90 (4.51±1.08)	2.90–5.36 (3.96±1.08)	3.80–7.00 (5.35±1.25)	2.50–4.64 (3.63±0.94)
Pat	2.30–4.64 (3.63±0.97)	2.10–4.30 (3.01±0.86)	1.80–3.50 (2.65±0.66)	2.20–4.20 (3.12±0.77)	1.80–3.20 (2.51±0.56)
Tib	2.70–4.90 (3.76±0.86)	2.10–3.70 (2.90±0.58)	1.75–3.90 (2.64±0.81)	2.80–4.80 (3.95±0.81)	1.90–3.20 (2.60±0.60)
Met	1.80–3.76 (2.64±0.80)	1.45–3.70 (2.55±0.79)	1.50–3.45 (2.59±0.71)	2.70–5.60 (4.11±1.15)	–
Tar	1.55–3.40 (2.33±0.64)	1.20–3.33 (2.13±0.74)	1.15–3.20 (2.24±0.65)	1.44–3.50 (2.59±0.65)	1.65–3.30 (2.56±0.61)
Total	12.00–23.10 (17.60±4.35)	10.20–20.90 (15.12±3.89)	9.50–18.20 (14.09±3.61)	13.41–25.10 (19.13±4.49)	8.30–14.20 (11.30±2.58)

#### Distribution and natural history.

**Figures 25–27.**
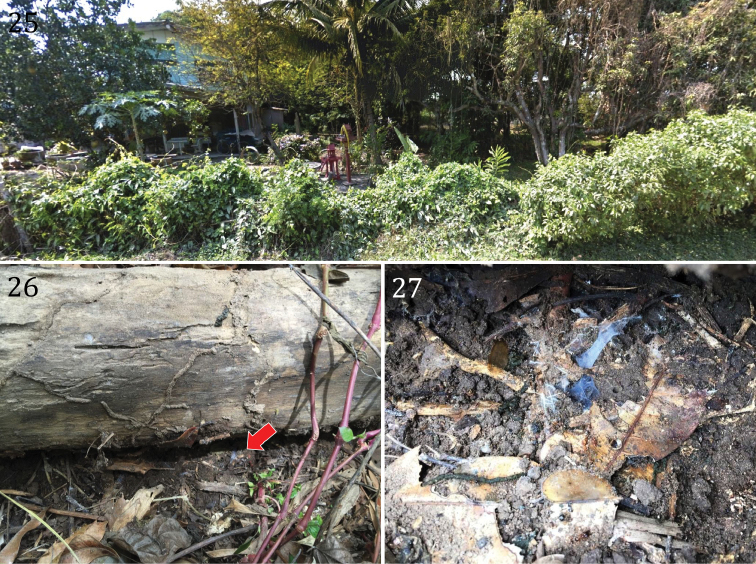
*Phlogiellus longipalpus* sp. n. Kamphaengphet province, Sai Thong Watthana. **25** locality where specimens are collected **26** habitat under log **27** subterranean system of silk tube retreats with litter.

Specimens were collected near villages in Lampang, Lamphun and Kampangpet provinces at approximately 200–500 meters in elevation. The habitat was disturbed by human activity and organic agriculture, including cultivation of mango, coconut, and bamboo (Fig. 25). Some specimens were collected from houses in the rainy season and others in shaded forest habitats; they appear to choose moist habitats. Some nests were built in colonies of termites or ants, which are used as prey (Figs 26–27). The nest consists of a shallow (1–2 cm deep) subterranean system of silken retreat tubes under stones or logs. One part of their web appeared to be used for accumulated prey scraps. Specimens from Saraburi province were collected in forest under rock and timber.

**Figure 28.**
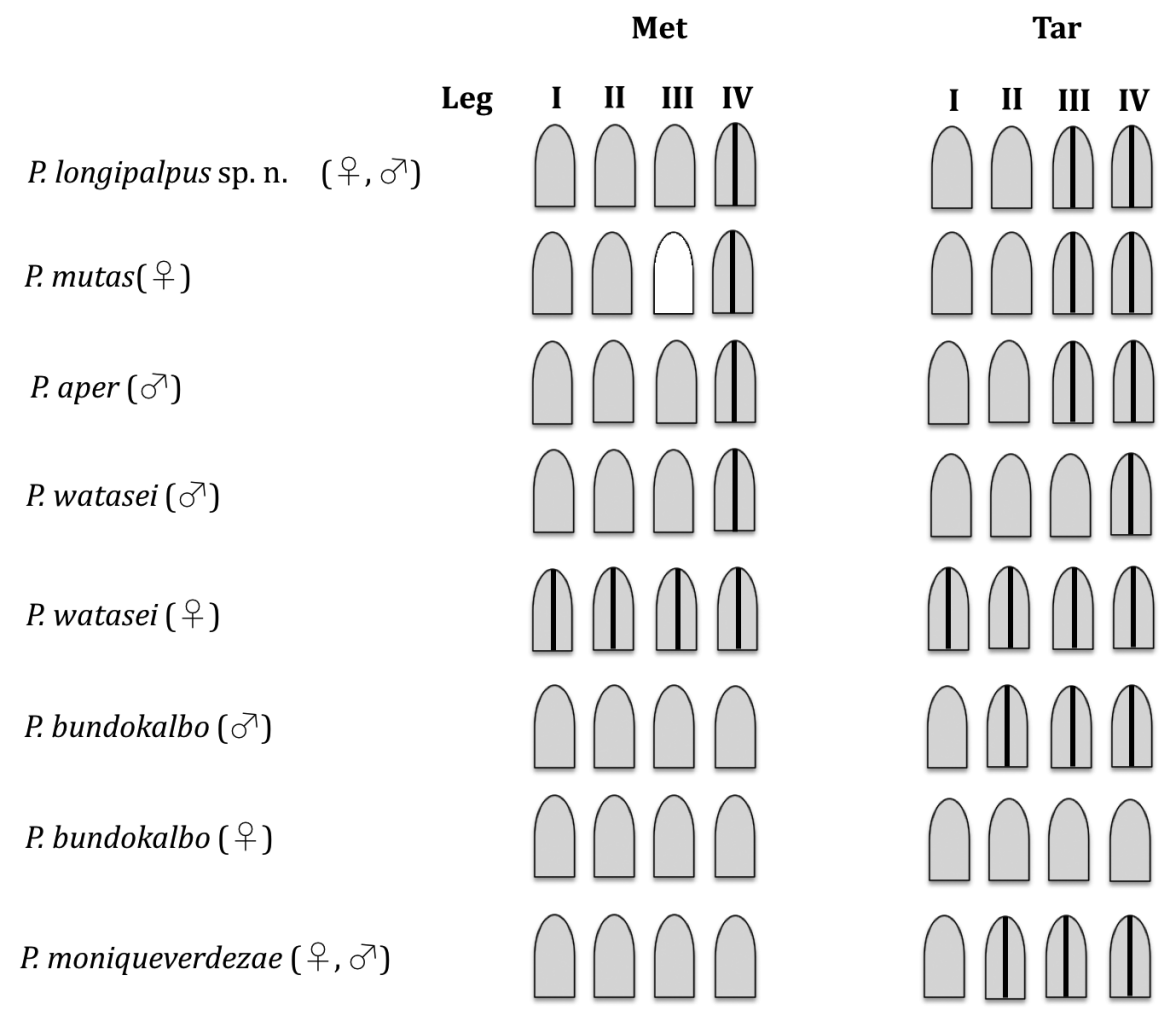
Metatarsal and tarsal scopae of legs I–IV of *Phlogiellus* lacking of maxillary lyra (*P. longipalpus* sp. n., *P. mutas*, *P. aper*, *P. watasei*, *P. bundokalbo*, and *P. moniqueverdezae*), indicating whether scopulae are entire, divided by rows of bristles or absent (grey – undivided, grey with black vertical line – divided, white – absent).

#### Remarks.

Nunn et al. (2016) recently revised *Phlogiellus* and gave very strong support for two synapomorphies of *Phlogiellus*: number of labial cuspules between 160–320, fewer than other selenocosmiine genera and very deep fovea. However, R. Raven (pers. comm.) pointed out that in Nunn et al. (2016), *P. pelidnus* Nunn et al., 2016 was described and shown to have more than 320 labial cuspules. This inconsistency of using the number of labial cuspules as a valid synapomorphic characters of *Phlogiellus* remains to be investigated. For our study, *P. longipalpus* sp. n. is placed in *Phlogiellus* as it has deep fovea, while the labial cuspule numbers are between 202–317 (average 281±42). *Phlogiellus longipalpus* sp. n. differs from *P. pelidnus*, *P. baeri* (Simon, 1877), *P. subinermis* (Giltay, 1934), *P. atriceps* Pocock 1897, *P. inermis* (Ausserer, 1871), *P. insulanus* (Hirst, 1909), *P. johnreylazoi* Nunn et al., 2016, *P. xinping* (Zhu and Zhang, 2008), *P. bogadeki* Nunn et al., 2016, *P. orophilus* (Thorell, 1897), and *P. obscurus* (Hirst, 1909) in lacking a maxillary lyra, a character it shares with *P. aper* (Simon, 1891), *P. brevipes* (Thorell, 1897), *P. watasei* (Kishida, 1920), *P. mutus* (Giltay, 1935), *P. bundokalbo* (Barrion and Litsinger, 1995), and *P. moniqueverdezae* Nunn et al., 2016. *P. longipalpus* sp. n. differs from other *Phlogiellus* species that lack a maxillary lyra by possession of a long embolus that is more or less 3 times longer than palpal bulb length (Suppl. material 1, Figs A1–A8) and long, uniquely shaped female receptacle (Suppl. material 1, Figs B1–B8). In addition, the pattern of tarsal scopula division illustrated in Fig. 28 can be used to distinguish mature specimens of *P. longipalpus* sp. n., *P. mutas*, *P. aper*, *P. watasei*, *P. bundokalbo*, and *P. moniqueverdezae*. This character cannot be used to diagnose *P. brevipes* (material not examined) or the female of *P. aper* (no specimens have been described) (Raven 2005, Guadanucci 2005, Nunn et al. 2016).

#### Distribution.

Thailand (Central and Northern).

## Supplementary Material

XML Treatment for Phlogiellus longipalpus

## Supplementary materials

Supplementary material 1 Supplemental figures Data type: Species figures This dataset is made available under the Open Database License (http://opendatacommons.org/licenses/odbl/1.0/). The Open Database License (ODbL) is a license agreement intended to allow users to freely share, modify, and use this Dataset while maintaining this same freedom for others, provided that the original source and author(s) are credited.Narin Chomphuphuang, Deborah Smith, Sitthipong Wongvilas, Varat Sivayyapram, Chaowalit Songsangchote, Natapot Warrit
