# Supplementary material for: New species of Southeast Asian Dwarf Tarantula from Thailand: Phlogiellus Pocock, 1897 (Theraphosidae, Selenocosmiinae)
Source: Zookeys. 2017 Jul 11;(684):57–73. doi: 10.3897/zookeys.684.12558 (PMC5523183; doi:10.3897/zookeys.684.12558)
Supplement: Supplementary material 1 — Supplemental figures [file zookeys-684-057-s001.docx]

**Supplemental Figure**


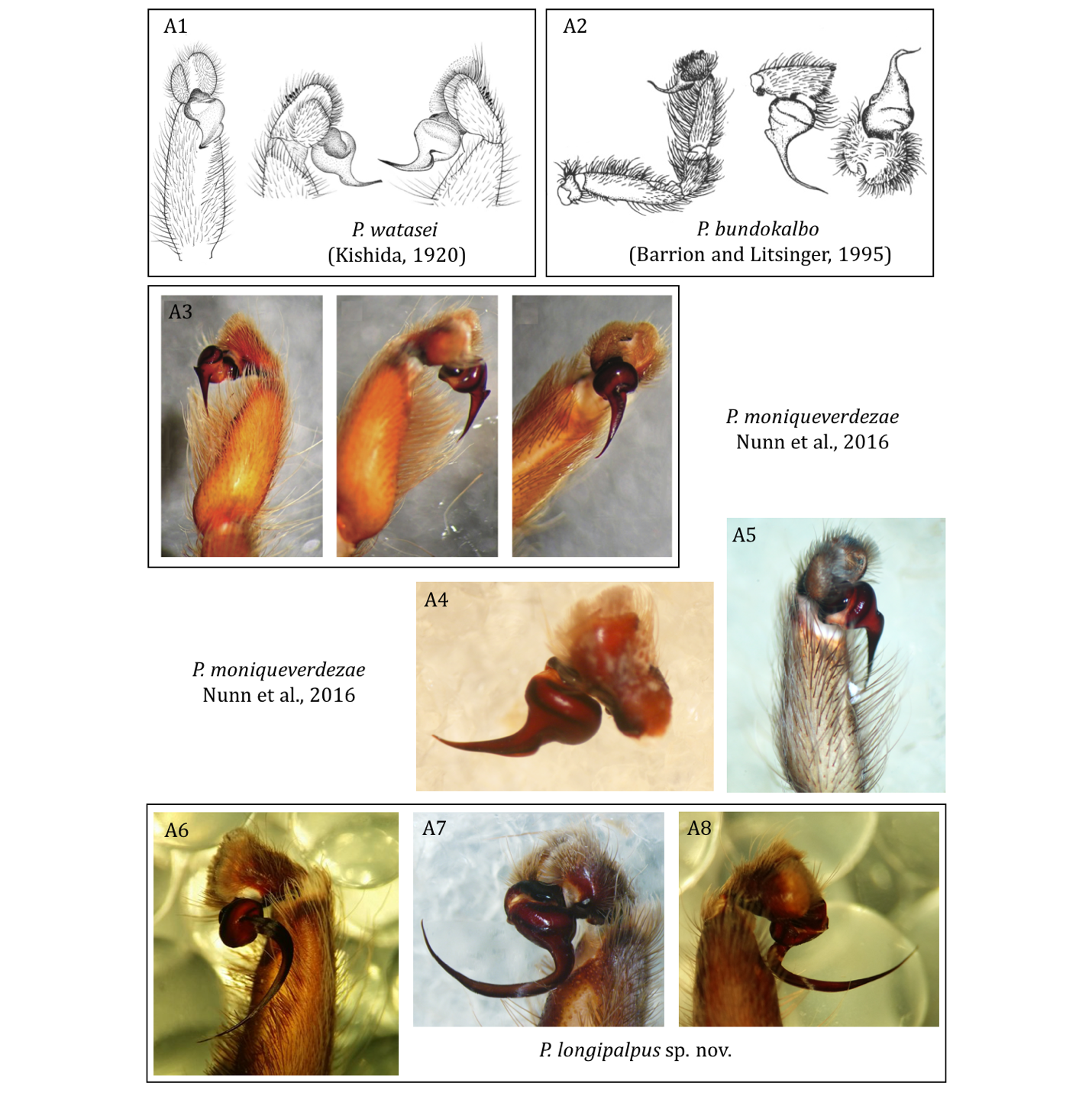


**Supplemental Figures A1–A8.** Male pedipalps of described *Phlogiellus* species (*Yamia* group), excluding *P. aper, P. brevipes* and *P. mutus* **Fig. A1.** *P. watasei*: ventral (left), prolateral (center), retrolateral (right) modified from Zhu and Zhang (2008) **Fig. A2.** *P. bundokalbo*: retrolateral (left), prolateral (center), ventral (right) modified from Barrion and Litsinger (1995) **Fig. A3.** *P.* moniqueverdezae: prolateral (left), prolateral (center), ventral (right) modified from Nunn et al. (2016) **Fig. A4–A5.** *P. moniqueverdezae* specimens collected by the authors (voucher specimens deposited at CUMZ): **A4**: CUMZ-T3-NA2M, left pedipalp prolateral view, **A5**: CUMZ-T9-COM, ventral view **Fig. A6–A8.** *P. longipalpus* sp. nov. left pedipalp **A6**: paratype; CUMZ-C4-NA4, retrolateral view, **A7**: holotype; CUMZ-C2-NA1, retrolateral view, **A8**: holotype; CUMZ-C2-NA1, prolateral view.


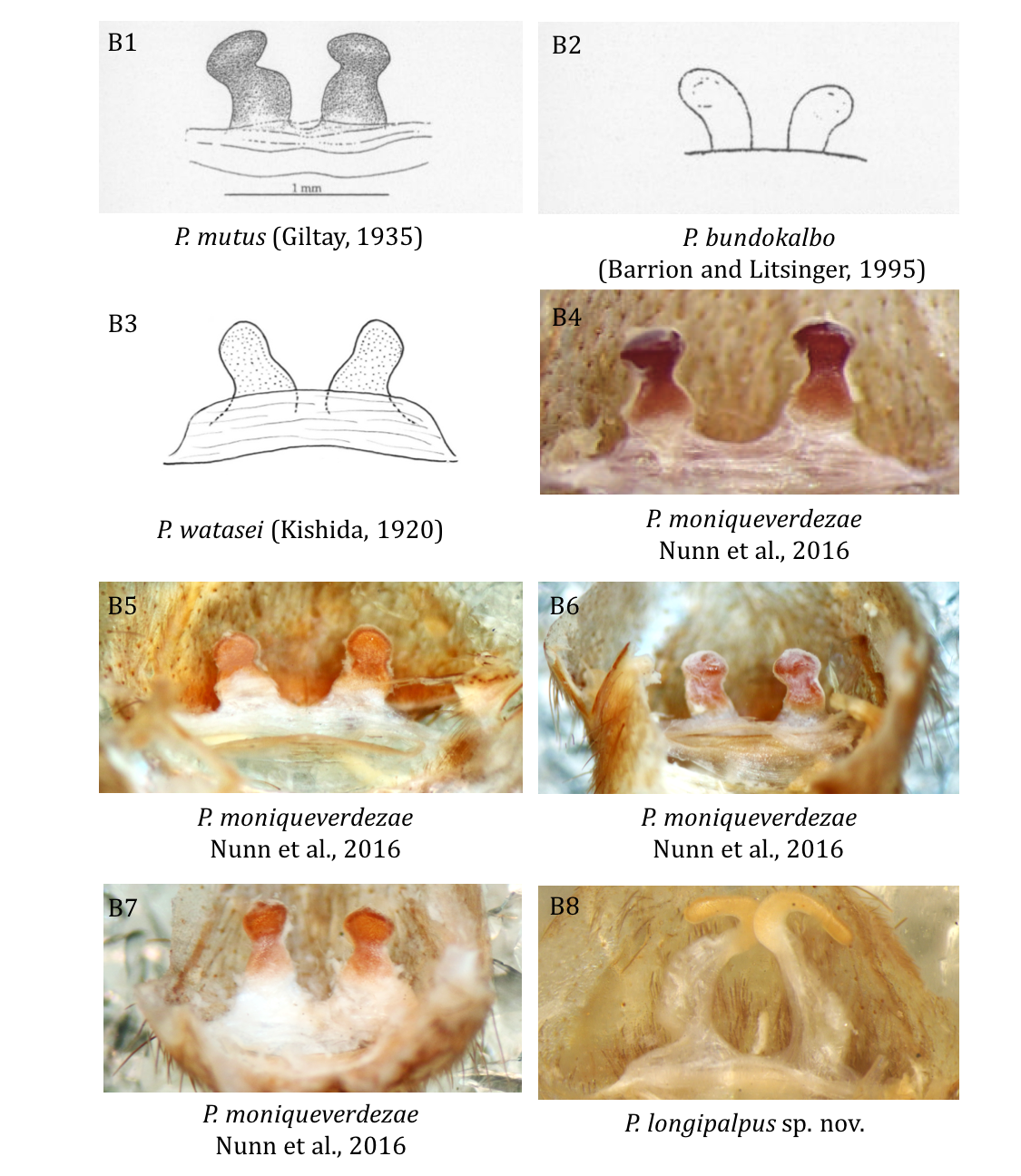


**Supplemental Figures B1–B8.** Spermathecae of described *Phlogiellus* species (*Yamia* group), excluding *P. aper* and *P. brevipes*, dorsal view **Fig. B1.** *P. mutus* modified from Schmidt (2010) **Fig. B2.** *P. bundokalbo* modified from Schmidt (2010) **Fig. B3.** *P. watasei* modified from Zhu and Zhang (2008) **Fig. B4.** *P. moniqueverdezae* modify from Nunn et al. (2016) **Fig. B5–B7.** *P. moniqueverdezae* specimens collected by the authors (voucher specimens deposited at CUMZ: **B5**: CUMZ-T3-NA5FM, **B6**: CUMZ-T3-NA3FM, **B7**: CUMZ-T10-COFM) **Fig. B8.** *P. longipalpus* sp. nov. (paratype; CUMZ-C4-NA4).
